# *Pseudogymnoascus destructans* growth in wood, soil and guano substrates

**DOI:** 10.1038/s41598-020-80707-1

**Published:** 2021-01-12

**Authors:** Jenny Urbina, Tara Chestnut, Jennifer M. Allen, Taal Levi

**Affiliations:** 1 Department of Fisheries and Wildlife, Oregon State University, 2820 SW Campus Way, Nash Hall, Corvallis, OR 97331 USA; 2 National Park Service, Mount Rainier National Park, Ashford, WA USA

**Keywords:** Microbiology, Ecology, Ecological epidemiology

## Abstract

Understanding how a pathogen can grow on different substrates and how this growth impacts its dispersal are critical to understanding the risks and control of emerging infectious diseases. *Pseudogymnoascus destructans* (Pd) causes white-nose syndrome (WNS) in many bat species and can persist in, and transmit from, the environment. We experimentally evaluated Pd growth on common substrates to better understand mechanisms of pathogen persistence, transmission and viability. We inoculated autoclaved guano, fresh guano, soil, and wood with live Pd fungus and evaluated (1) whether Pd grows or persists on each (2) if spores of the fungus remain viable 4 months after inoculation on each substrate, and (3) whether detection and quantitation of Pd on swabs is sensitive to the choice to two commonly used DNA extraction kits. After inoculating each substrate with 460,000 Pd spores, we collected ~ 0.20 g of guano and soil, and swabs from wood every 16 days for 64 days to quantify pathogen load through time using real-time qPCR. We detected Pd on all substrates over the course of the experiment. We observed a tenfold increase in pathogen loads on autoclaved guano and persistence but not growth in fresh guano. Pathogen loads increased marginally on wood but declined ~ 60-fold in soil. After four months, apparently viable spores were harvested from all substrates but germination did not occur from fresh guano. We additionally found that detection and quantitation of Pd from swabs of wood surfaces is sensitive to the DNA extraction method. The commonly used PrepMan Ultra Reagent protocol yielded substantially less DNA than did the QIAGEN DNeasy Blood and Tissue Kit. Notably the PrepMan Ultra Reagent failed to detect Pd in many wood swabs that were detected by QIAGEN and were subsequently found to contain substantial live conidia. Our results indicate that Pd can persist or even grow on common environmental substrates with results dependent on whether microbial competitors have been eliminated. Although we observed clear rapid declines in Pd on soil, viable spores were harvested four months after inoculation. These results suggest that environmental substrates and guano can in general serve as infectious environmental reservoirs due to long-term persistence, and even growth, of live Pd. This should inform management interventions to sanitize or modify structures to reduce transmission risk as well early detection rapid response (EDRR) planning.

## Introduction

Emerging fungal pathogens have become a major conservation issue for wildlife^1–3^. Fungal pathogen transmission occurs through diverse pathways causing opportunistic infections such as aspergillosis^4^, cryptococcosis^5^ and mucormycosis^6–8^, and endemic infections with indirect transmission from the environment such as blastomycosis^9,10^ and histoplasmosis^11^. Additionally, there are zoophilic pathogens with near-direct transmission such as the chytrid fungi *Batrachochytrium dendrobatidis*^12^ and *Batrachochytrium salamandrivorans*^13^ that cause chytridiomycosis. These highly variable modes of transmission arise because fungi readily survive outside of hosts and can persist, or even grow, on environmental substrates.

Although hosts are typically central to pathogen transmission, the epidemiological triangle reminds us of the importance of the environment on transmission dynamics^14,15^. In the traditional triad, the physical environment can modify the response of both the pathogen and the host^16,17^. The presence and types of fomites can influence dispersal of pathogens and disease dynamics^18,19^. Therefore, the role of substrates is significant in situations where viable pathogens from an environmental substrate can re-infect hosts or remain as an environmental reservoir^20–22^. Understanding the growth and survival of pathogens on environmental substrates is critical to inform management interventions that might sanitize or modify structures to reduce transmission risk^23,24^. Obtaining a better insight about links among substrates, pathogen and hosts will improve our knowledge of pathogen transmission and inform actions and strategies for disease mitigation.

One of the most important fungal pathogens for wildlife is *Pseudogymnoascus destructans* (Pd), which causes white-nose syndrome (WNS) disease in bats. The first evidence for the emergence of this disease in North America was reported in Albany, New York in 2006^25,26^. By 2009 WNS had been reported from four species including little brown bat (*Myotis lucifugus*), northern long-eared (*M. septentrionalis*), big brown (*Eptesicus fuscus*) and tricolored bats (*Perimyotis subflavus*). Since then, WNS has been reported in nine additional species within the genus *Myotis*. WNS causes mortality in bats by damaging skin and disrupting critical physiological processes that can result in starvation or dehydration^27–29^. This disease has been implicated in the unprecedented decline of previously abundant, widely distributed, and healthy bat populations^30–33^, and it is estimated to have killed more than 6 million bats^34^. To date, eight bat species and subspecies from the genera *Corynorhinus* (4),* Lasionycteris*,* Lasiurus*,* Tadarida* and *Myotis* (http://www.whitenosesyndrome.org, accessed April 13, 2020) have been tested positive for Pd according to qPCR results, without diagnostic signs of the disease documented in the tested individuals.

Pd can be transmitted directly among bats^35^ or indirectly via environmental substrates^36^. Indirect transmission of Pd is facilitated by long survival times^37^ and persistence outside of its optimal growth temperature^38^. The prevalence of Pd in hibernacula remains stable or slightly increases through time after the initial detection of the pathogen^22^. Pd is persistent in hibernacula in the United States while in Eurasian hibernacula the prevalence and pathogen loads decrease due to a seasonal decay of Pd during summer that leads to delayed infection, shorter period of Pd growing on bats and low loads^22^. Although transmission of Pd has been linked to colony size and clustering^32^, the role played by particular substrates as environmental reservoirs on disease impacts is only partially understood^39^.

We conducted an experimental study of Pd growth and survival on guano, soil, and plywood, which are typical environmental substrates present in bat hibernacula or roosting locations. We assessed whether Pd would grow on guano to determine whether guano could be an effective environmental reservoir for Pd and a transmission pathway to susceptible bats. We hypothesized that guano might supply ample nutrients for growth, but this growth might be inhibited by fecal bacteria. Therefore, we assessed Pd growth and viability on both non-sterile and autoclaved guano. We tested Pd growth and persistence on plywood because this is a common substrate for bats found in structures made by humans particularly artificial roost habitats such as bat houses. Based on unexpectedly low Pd concentrations detected in this substrate, we additionally compared the efficiency of two commonly used DNA extractions kits. Finally, we tested soil because it is a common substrate beneath hibernacula and roost sites. We inoculated each substrate with a known amount of *P. destructans* conidia to answer whether (1) Pd grows or persists on all the substrates, (2) there is a difference in the growth rates of Pd among different substrates, and (3) whether conidia retrieved from each substrate were still viable after the 64-day experiment.

## Methods

We characterized the growth of Pd in four substrates (1) autoclaved guano, (2) fresh guano, (3) soil and (4) wood. We collected fresh guano of *Myotis lucifugus* from different piles in the attic of a storage barn in Corvallis, Oregon, USA. Half of the guano collected was autoclaved for 60 min at 121 °C and at least 20 psi (Consolidated, MA, USA) and the other half was not sterilized. We used commercial garden soil Proven Winners (all-purpose type) as our soil substrate. As previous studies have already established the viability of Pd on sediments^40,41^, we assessed the growth/decay rate in only non-autoclaved soil similar to field conditions. Finally, we autoclaved (to control for the presence of other fungi and bacteria) square pieces of plywood (5.08 cm side, ~ 3 mm thick) as our wood substrate to evaluate if Pd can persist on this substrate and to determine whether the substrate itself was capable of supporting Pd.

**Figure 1:**
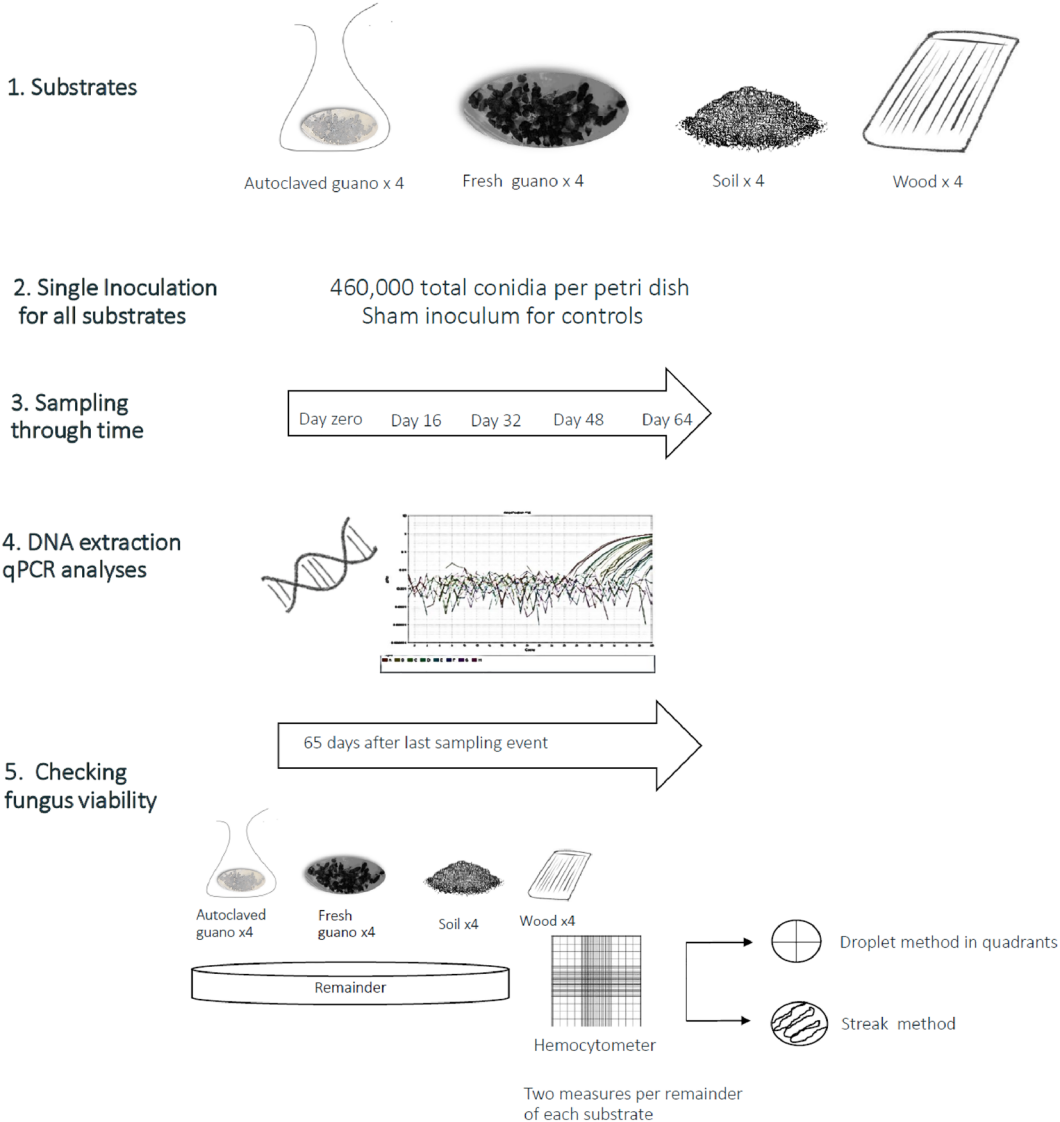
Graphic summary of the experimental design and methods we used during our experiments.

We prepared the inoculum by resuspending, counting, and harvesting Pd conidia from cultured plates following standard methods^35^. Cultures of the Pd type strain (American Type Culture Collection ATCC MYA-4855) were maintained for 30 days on Sabouraud Dextrose Agar (SDA) with gentamicin at 9 °C. We prepared a suspension by flooding plates with phosphate—buffered saline solution containing 0.5% Tween (PBST) and counted the conidia using a hemocytometer. We inoculated four replicates of autoclaved guano, fresh guano, wood and soil held in individual petri dishes by pipetting 1 ml of inoculum with a total estimated dose of 4.6 × 10^5^ Pd conidia on top of 1.06 (± 0.0066) grams of substrate. A similar dose (5 × 10^5^) produced WNS infection and induced behavioral changes in bats^35,42^. We gently moved the samples to evenly distribute and spread the inoculum on the guano and soil substrates. Inoculation of the plywood pieces was done by dividing the piece of wood into five equal parts that were assigned a random order to be sampled. We inoculated each part of the plywood by pipetting 5 drops of 40 μl per part (200 μl), so the total piece of wood had the same amount of conidia as the other substrates (Fig. 1). We added one negative control replicate per substrate that was inoculated as previously described with PBST without conidia. We used SDA plates with Pd growing as positive control.

We performed the first sampling event (time zero) immediately after adding the Pd inoculum to all the substrates. We collected 0.18 g (± 0.05) grams of autoclaved guano, fresh guano and soil to quantify the amount of Pd for each substrate replicate petri dish. For the wood sampling, sterile rayon swabs (MVE, Medical Wire, UK) were rolled into the length of the plywood within a row allocated for each sampling event to avoid swabbing the same portion of wood more than once.

We continued collecting samples from each substrate at four regular intervals every 16 days until day 64. All petri dishes holding the substrates were kept in a refrigerator at 9 °C without particular light/dark cycle and were organized randomly. For guano and soil, we collected an average mass of 0.23 (± 0.08) grams. Samples were stored in vials at − 20 °C prior to DNA extraction for no longer than 2 weeks.

We performed DNA extractions from autoclaved guano, fresh guano, and soil using the DNeasy Powerlyzer Powersoil Kit (QIAGEN, MD, US), which includes inhibitor removal steps^44^. Replicate swab samples from plywood were extracted using the PrepMan Ultra Sample Preparation Reagent (Applied Biosystems, Inc, Foster, CA, USA) and DNeasy Blood and Tissue Kit (QIAGEN, MD, USA), which are commonly used for detection of Pd from swabs^21,22,45,46^. PrepMan Ultra Reagent is a common choice due to its low cost and ease of use. The additional purification steps with the QIAGEN DNeasy Blood and Tissue Kit may reduce PCR inhibition but is 3 times more expensive than PrepMan (PrepMan $0.735/sample, DNeasy $2.94/sample). We compared the efficiency of these methods by evaluating the detection (presence/absence of pathogen) and yield of DNA after the first sampling event through the end of the experiment. We began this comparison after the first sampling event because initial results using the PrepMan Reagent returned less DNA than expected. All samples were extracted individually after each sampling event and one extraction blank was included at each sampling event.

After DNA extraction, we quantified Pd using a probe-based qPCR assay targeting the intergenic spacer region (IGS) of the fungus^47^. Our reactions contained 12.5 μl of TaqMan Environmental MasterMix 2.0 (Life Technologies, Carlsbad, CA), forward primer nu-IGS-0169-5′Gd and reverse primer nu-IGS-0235-3′Gd at a final concentration of 400 nM, TaqMan FAM-labeled probe at a final concentration of 200 nM and 5 µl of DNA template^47^. We used an ABI PRISM 7500 Fast real-time PCR system (Applied Biosystems, Foster City, CA) with the following cycling conditions: initial activation 95 °C for 10 min; denaturation 95 °C for 15 s, annealing and extension 60 °C for 60 s with a total of 40 cycles. We used a 4-point standard curve in triplicate from 10 to 10,000 fg of genomic DNA (ATCC MYA-4855™) to quantify the amount of DNA in each sample. We analyzed all samples in triplicate and they were reported as positive if 2 out of the 3 wells amplified within 40 cycles.

We harvested conidia from the remaining material from each substrate 51 days after the last sampling event, which corresponds with 115 days post inoculation. We soaked the substrate material in 5 ml of PBST for 15 min. We used a binocular compound microscope (40 × magnification, Model B120C, AmScope) to count the number of conidia in two replicates of the resuspended substrate using a hemocytometer. To confirm conidia viability, we plated 10ul of the harvested liquid using the droplet method in two SDA petri dishes (150 mm × 15 mm) divided into four quadrants. We also used the streak method to plate two additional petri dishes for each sample using a sterile cotton swab that was submerged in the harvesting liquid. We visually checked and photographed the plates to assess whether harvested spores were viable two weeks later (i.e. 65 days after the last sampling event, which corresponds with 129 days post inoculation).

We used standard protocols for decontamination of prepared suspensions of bleach and ethanol as established by the National White-nose syndrome decontamination protocol v09.13.2018^43^. All material was additionally autoclaved before being discarded.

### Data analysis

For each substrate, we evaluated evidence for Pd growth or decay using linear mixed models to model log-10 Pd DNA quantity as a function of days since inoculation using each sampling event per petri plate as random effect to account for the non-independent qPCR replicates nested within each DNA extraction. We added a positive constant value (141.75 fg), the minimum amount of Pd detected, to handle loads of Pd reported as zeros. We interpreted an increase in DNA concentration as evidence of Pd growth and stable levels as evidence of Pd persistence. Detection of Pd DNA does not necessarily imply that Pd is alive and pathogenic^39^. For each day corresponding to a sampling event (i.e. every 16 days since inoculation) we included data from 4 replicate inoculations each with 3 qPCR replicates per substrate type (Fig. 1). We additionally used the regression coefficients of this model to project estimated untransformed Pd DNA quantities through time. For these analyses, we only included DNA quantified after extraction using QIAGEN kits (i.e. excluding DNA quantities derived from the the PrepMan Ultra extraction kit used for wood swabs).

We compared the DNA yield from swabs of plywood extracted with the commonly used PrepMan Ultra and Qiagen Blood Tissue Kits using a linear model for each sampling event with extraction method as a factor. This result excludes data from the first sampling event that was extracted only with the PrepMan Ultra kit. Statistical analyses were done in R (Version 3.4.4, R Core Team 2017). All procedures were approved by the Institutional Biosafety Office (IBC) at Oregon State University (IBC tracking number 4218).

## Results

**Figure 2:**
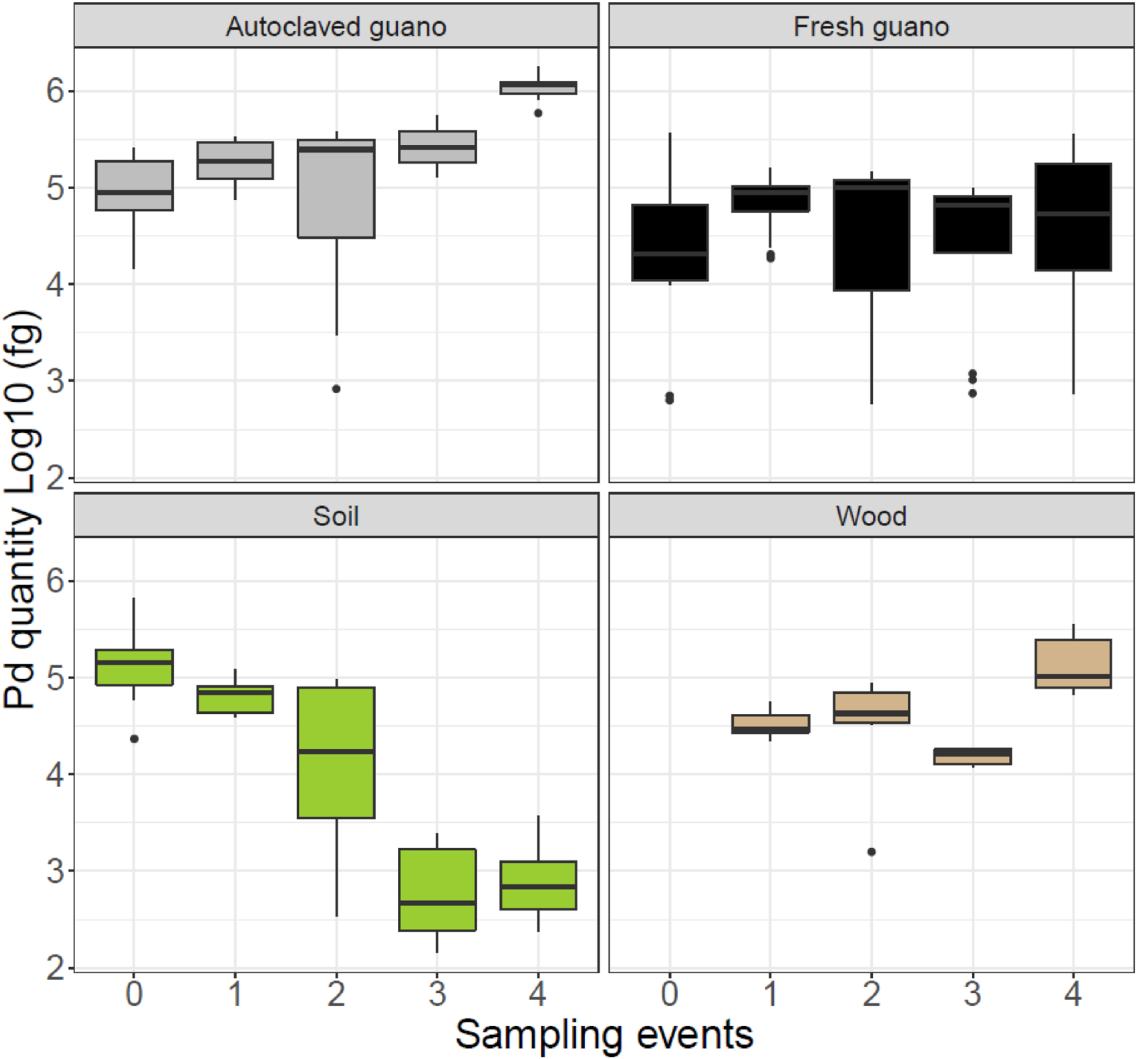
Pd fungus growth on different substrates over 64 days for five different sampling events (Day 0, 16, 32, 48 and 64). To facilitate comparison with the same extraction kit, we only present data for wood corresponding to values obtained using the QIAGEN Blood and Tissue extraction Kit. We discovered substantially lower yield with the Prepman Ultra Reagent after the first sampling event and initiated sampling with both Prepman and QIAGEN kits for all subsequent sampling events.

*Pseudogymnoascus destructans* was detected in all substrates during all our sampling events (Fig. 2 and Table S1). In autoclaved guano, the quantity of Pd detected increased over time (β_autoclaved_ = 0.014; P = 0.003). There was a tenfold increase in the amount of Pd detected from the experimental setup to the last sampling event. Pd remained stable in fresh guano (β_fresh_ = − 0.0003; P = 0.007) but decreased substantially in soil (β_soil_ = − 0.03; P = 0.004). We observed a significant increase on wood (β_wood_ = 0.009; P = 0.009) despite the most Pd DNA detected during the final sampling session. This effect was not stronger due to low Pd DNA detected during the third sampling event for unknown reasons (Fig. 2). All controls were negative for Pd amplification.

**Figure 3:**
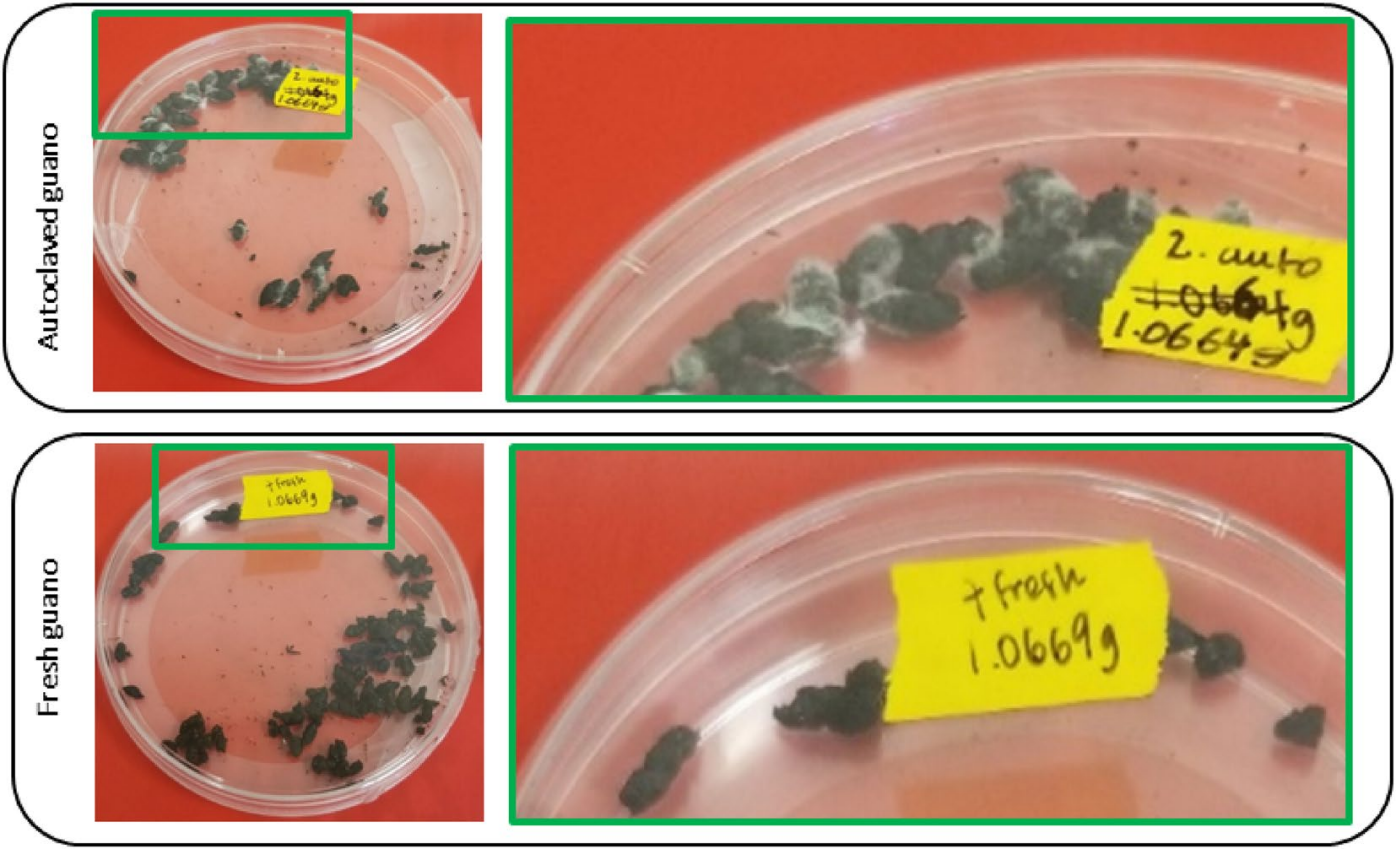
Plates of autoclaved guano and fresh guano pellets 48 days after inoculation. Top row shows visible growth of *Pseudogymnoascus destructans* on autoclaved guano pellets compared to bottom row without visible growth on fresh guano pellets.

*Pseudogymnoascus destructans* grew optimally in autoclaved guano. After 12 days, it was possible to see mycelial mat on top of the autoclaved pellets but no growth was visible on fresh guano (Fig. 3). Although growth through time was detected by qPCR in wood, there were no visible mycelia on top of this substrate during the experiment. Similarly, in soil, there was no visually noticeable growth of the fungus.

**Figure 4:**
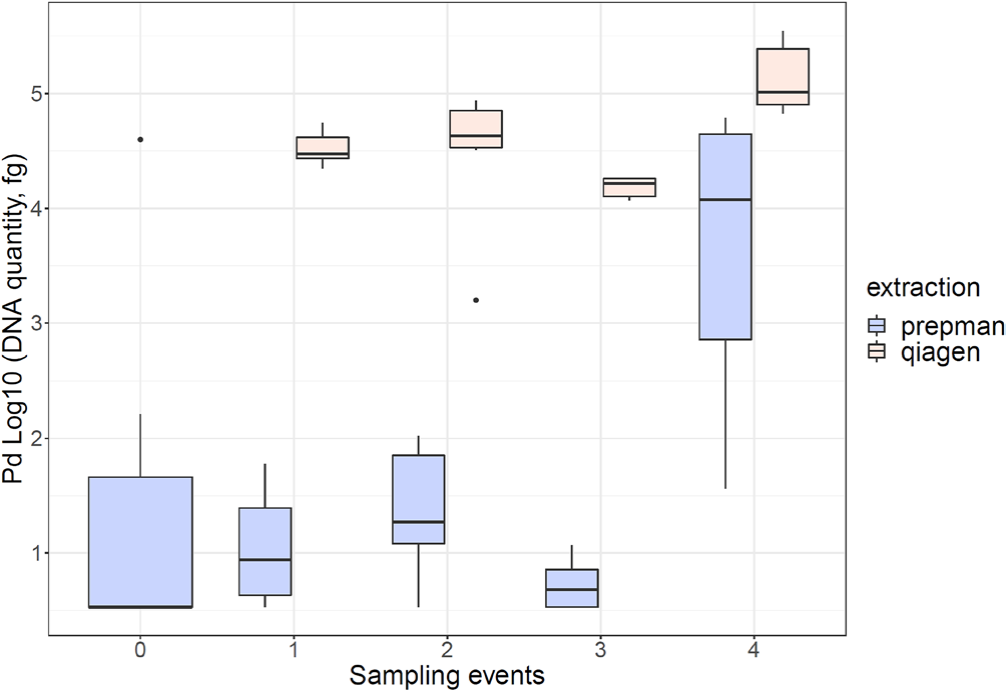
Fungal loads recovered from plywood using either PrepMan Ultra Reagent or QIAGEN DNeasy Blood and Tissue Kit for DNA extraction.

The quantity of fungus detected in wood varied according to the extraction protocol used. For all sampling events, the average amount of DNA detected was higher when using the Blood and Tissue Kit (QIAGEN). For the first sampling event, we detected more DNA when using the QIAGEN Kit (β_1_ = 34,918.80, P < 0.00006) in contrast to Prepman Ultra (16.45 fg). Similarly, the Pd quantities were higher for the second (β_2_ = 47,898.19, P = 0.004) and third sampling event (β_3_ = 15,553, P < 0.00000023) after using the QIAGEN Kit in comparison to Prepman Ultra (39.79 fg and 2.60 fg). During the last sampling event, the amount of Pd detected was high in comparison to previous events after using Prepman Ultra (24,435 fg), however the QIAGEN Kit detected a higher amount (β_4_ = 144,058, P = 0.02) (Fig. 4).

**Figure 5:**
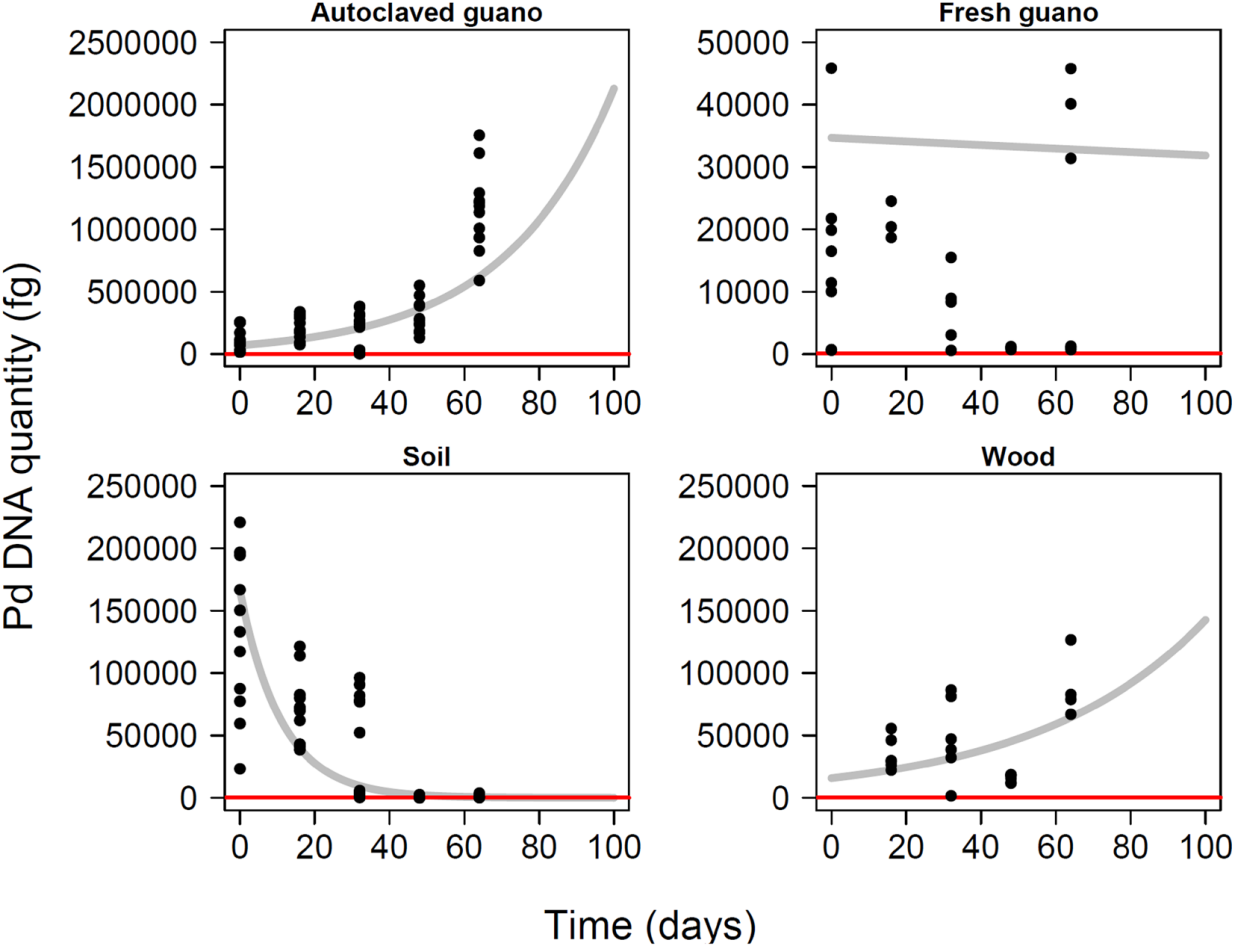
Model projections for quantity of *Pseudogymnoascus destructans* during 100 days in four different substrates with experimental data overlapped. Red line indicates the lowest amount of Pd detected during this experiment.

The projections for fungus growth extrapolated for 100 days post inoculation highlight the trajectory toward high levels of Pd in autoclaved guano, stability in fresh guano, undetectable levels in soil, and growth in wood (Fig. 5).

**Figure 6:**
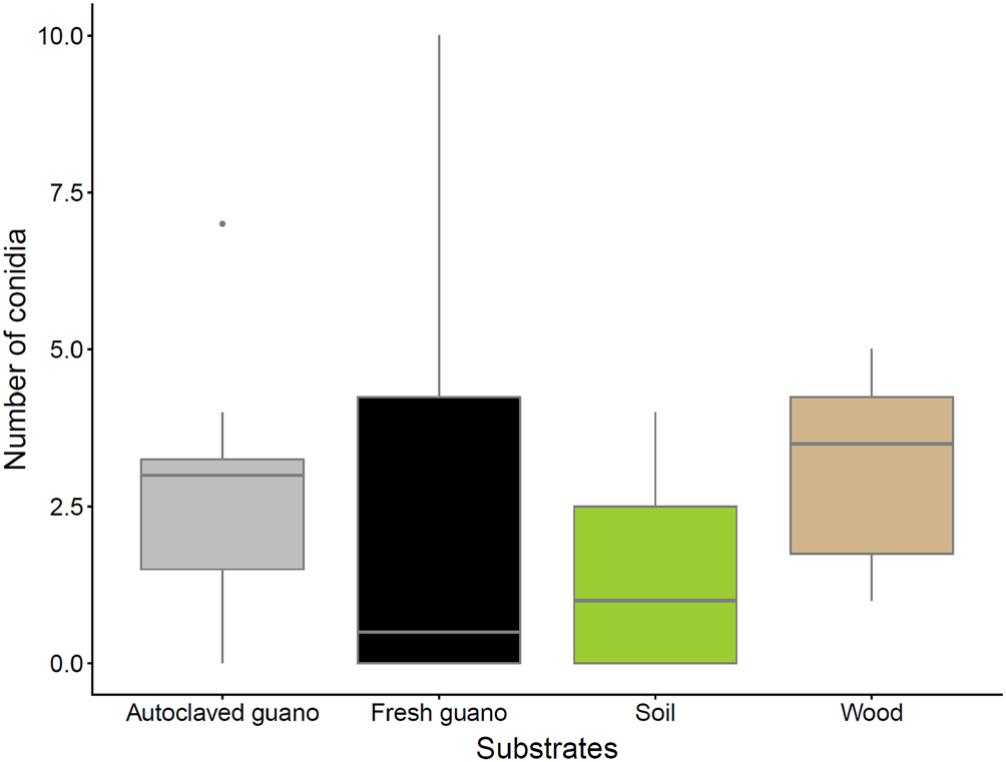
Number of conidia counted per substrate 51 days after termination of the experiment to check viability in SDA cultures (115 days post inoculation).

Counts of conidia 51 days after finishing the experiment were low for all the substrates. Wood had the highest average number of conidia (mean 3.12 ± SD 1.64), followed by autoclaved guano (mean 2.75 ± SD 2.25), fresh guano (mean 2.5 ± SD 3.62) and soil (mean 1.5 ± SD 1.69 (Fig. 6).

**Figure 7:**
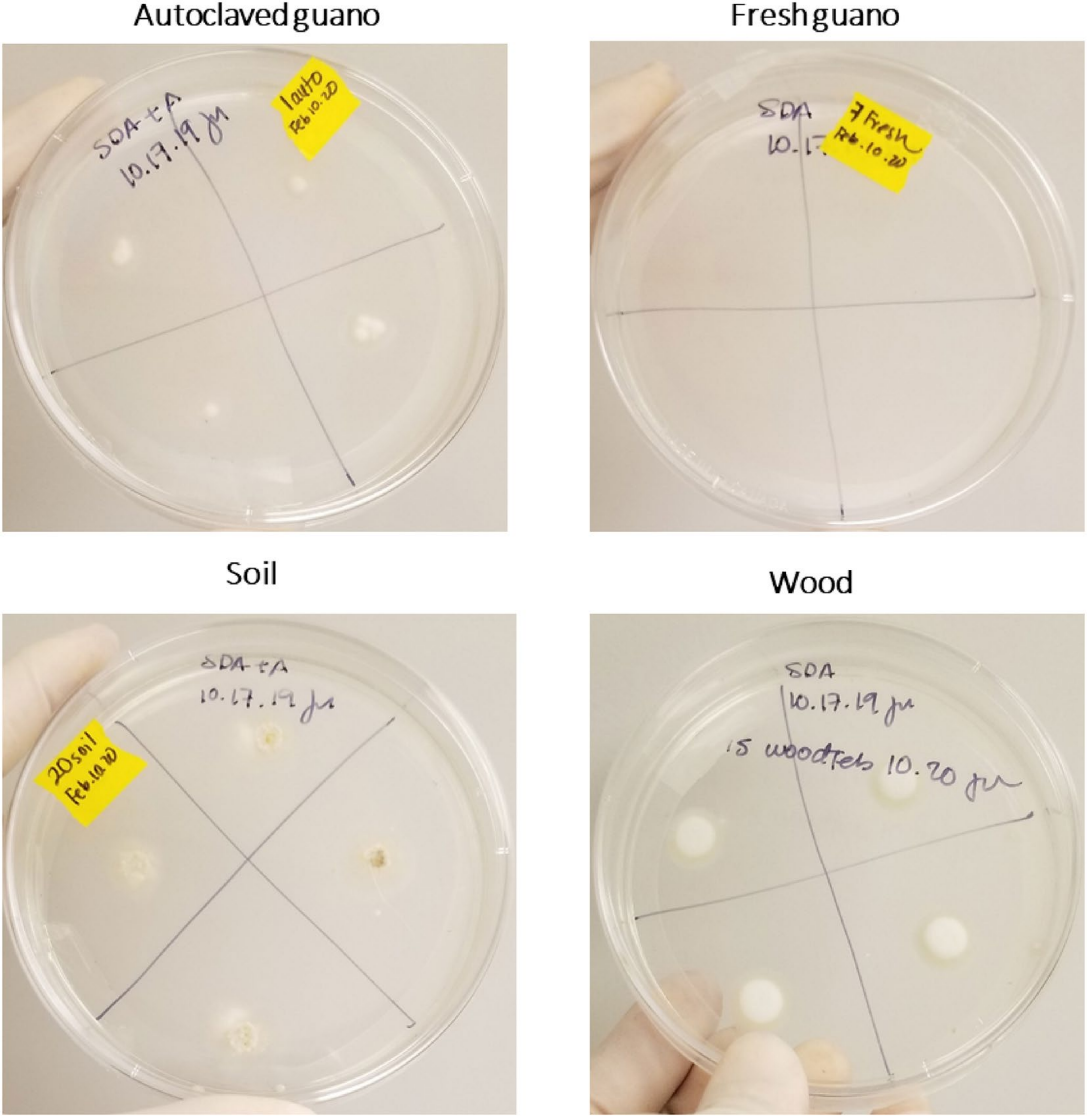
Growth of Pd in SDA plates 14 days after plating 10 μl of harvesting liquid obtained from leftovers of the different substrates evaluated.

Pd grew in all four quadrants of SDA plates inoculated with 10 μl of the harvesting liquid obtained from wood, soil and autoclaved guano. No growth was evident in plates inoculated with material from fresh guano (Fig. 7). There was no growth of Pd in plates inoculated with the sham control.

## Discussion

The increase of invasive fungal infections worldwide requires accurate and efficient identification of pathogens and their viability. Detection of *Pseudogymnoascus destructans* through monitoring and surveillance is performed by detecting DNA extracted from swabs or environmental samples using qPCR. However, samples can be qPCR positive either because of extracellular DNA^39^, DNA from dead cells, or DNA from live fungus, but only live fungus is infectious. Therefore, assessing the growth and viability of pathogens such as Pd on different substrates can provide insight about the role of environmental substrates as reservoirs to inform management of potential fomites implicated in the spread of the disease.

We have demonstrated that live fungus can persist for long periods in the absence of a host and grow on environmental substrates for over 2 months and retain infectious conidia for at least four months. The differential growth and survival of this pathogen on the substrates we evaluated can be explained by the interplay between medium composition, nutrient availability^48^, and microbial antagonists^49–51^. Pd grew substantially on autoclaved guano as reflected by a tenfold increase in DNA quantity detected over the course of the study (Fig. 2) as well as visible fungal growth (Fig. 3). The autoclaved guano depleted of microbial organisms presumably allowed for substantial Pd growth by providing nutrients in a substrate without microbial competitors. In contrast, Pd persisted but did not grow detectably in fresh guano over the full 64-day experiment and harvested spores did not germinate. The diversity of bacterial communities in fresh guano includes Phyla such as Actinobacteria^52^ and members of the families Enterococcaceae, Baciillaceae and Enterobacteriaceae^53,54^. Actinobacteria play a role in the decomposition of organic material. *Enterococcus* species are reported to inhibit hyphal morphogenesis and virulence of fungus^55,56^, while *Bacillus* and *Enterobacter* species are reported to control fungal growth by synthesis of hydrolytic enzymes and production of antifungal compounds e.g., bacterial volatile compounds^57^, and inhibiting sporulation^58^ respectively. As similar mechanisms could be affecting the growth of Pd in fresh guano, we recommend that future studies of Pd viability evaluate microbial competition on Pd persistence and transmission. These studies can benefit from the use of culture media types with a better resemblance to the complex nutrient of the host^38^. We observed long-term persistence of Pd on sterile plywood. We sterilized plywood to determine whether the woody substrate was a suitable medium for growth while controlling for extant microbial communities that might be present. Such microbial communities on non-sterile plywood might also inhibit Pd growth, but we expect this effect to be small relative to fresh guano. In contrast, DNA concentrations of Pd in soil declined ~ 60-fold over the course of the experiment. This could be related to the presence of soil fungi such as *Mortierella* spp. and *Mucor* spp., which are fast-growing and potentially able to outcompete Pd^48^. The presence of soil fungi growing on our SDA plates in addition to Pd provides some support for this hypothesis. Future studies can benefit from evaluating soils found in roosting places and sediments from caves to understand Pd growth, persistence and viability in these substrates.

Although we counted conidia from resuspension prepared from leftover material of each substrate, Pd was only viable in culture for autoclaved guano, soil and wood (Fig. 7). This is unsurprising for autoclaved guano, which exhibited visible Pd growth and a large increase in Pd DNA concentration (Figs. 2, 3). However, conidia were collected from soil and readily germinated despite rapidly declining Pd DNA concentrations below the limit of detection (Figs. 2, 7). We were also surprised to harvest the most conidia from wood and to observe the most visible growth in culture (Figs. 6, 7). Although harvesting seemingly viable conidia from fresh guano, we did not detect growth of Pd after inoculating SDA plates with these conidia. We rule out the number of viable conidia as a potential explanation as the number obtained was similar between autoclaved (2.75 conidia) and fresh guano (2.5 conidia). This lack of growth can potentially be explained by effects produced by bacteria action such as production of volatile compounds that can reduce the growth from conidia and mycelial extension of Pd^59,60^. Alternatively, fungal spores are able to enter into exogenous dormancy to later germinate and grow when the effect of an unfavorable chemical or physical condition stops^61,62^, which could explain these results. Therefore, although we did not see germination, we cannot assume that conidia will not germinate at a later time when conditions become more favorable. Recently, Pd was reported growing at higher temperatures (24 °C, 30 °C and 37 °C) outside of its optimal temperature growth^38^ exemplifying the ability of this pathogen to thrive in different conditions. Also, Pd has been reported as viable 238 days after inoculation in sediments as flood debris with the growth of the pathogen being influenced by geochemistry and the total amount of organic carbon available^41^. Physical and chemical characteristics of the evaluated substrates can directly or indirectly affect the growth or persistence of Pd through time. Changes in relative humidity, nutrient content, pH or temperature can trigger germination in ascomycete fungi^63^. The vegetative growth of Pd increase with high levels of relative humidity while the production of conidia is not affected by relative humidity^64^. Pd is able to grow in a broad pH range between 5 and 11 with substrates as dead fish *Poecilia* sp., insect (*Locusta migratoria*), mushrooms (*Lentinula edodes*) and demineralized exoskeletons of shrimp (*Pleoticus muelleri*) appropriate for the fungus to germinate and grow^65^. Also, a range of chemical compounds like essential oils^66,67^, volatile organic compounds^60^ can also affect the growth and persistence of Pd.

The viability of Pd on environmental substrates suggests that many exposed substrates could be infectious to bats. The viability of Pd on plywood could be an indication that human made structures could be more important than bat guano for disease spread, particularly if the fungus invades and persists in the wood substrate. Importantly, our results suggest that sampling wood surfaces with swabs and extract DNA using Prepman Ultra will result in under-detection of Pd from buildings and structures such as attics and bat boxes. It is plausible that hot summer temperatures could partially sterilize guano that may then serve as an efficient medium for Pd growth in fall and winter. Researchers who come into contact with environmental substrates, including guano, should take great care not to spread Pd to new environments or bats. Risk analysis for WNS in Australian bats has accounted for this possibility with specific policy to implement controls to identify and decontaminate fomites on environmental substrates to reduce the spread of Pd^68^. We recommend updating and clarifying existing disinfection protocols to ensure effective methods for decontamination, including sites that are outside of caves and enclosed spaces where guano may accumulate, such as in building attics and below a constructed bat house. Evaluations of existing chemical treatments to determine what dose and substance constitutes an efficient treatment as sporicidal (killing spores) or fungistatic (inhibition of fungal growth) will ensure complete decontamination and prevention of spreading Pd. We suggest additional experimental work to evaluate the effectiveness of current decontamination protocols, so there are assurances that field and laboratory decontamination protocols can minimize the risk of humans-cause spread of the pathogen.

Our experiment comparing DNA concentrations obtained through the two methods of extraction (PrepMan Ultra Reagent and QIAGEN) has substantial implications for surveillance needing to sensitively detect Pd. Use of the more affordable PrepMan extraction protocol is substantially more likely to lead to false negative results. Surveillance in locations with unknown infection status should prioritize high extraction efficiency given that Pd prevalence at these locations is expected to be low. This is also the case for surveillance conducted among seasons to allow for the detection of minimum changes in pathogen load on environmental reservoirs of Pd. In cases when infection is already high, both methods may work adequately for detection, but DNA concentrations determined with PrepMan will be estimated with lower precision. To ensure the highest level of testing sensitivity, practitioners should consider using the QIAGEN protocol, especially in locations where Pd and WNS is not yet known to occur and where a positive detection may result in a management action such as a cave closure. This testing sensitivity could facilitate management actions including clearer communication of the presence and spread of Pd, leading to more assertive action plans for bat conservation.

This research contributes important information to the understanding of the interaction between environmental reservoirs and Pd, which has been previously overlooked, as research in WNS has focused mainly on the pathogen-host relationship. This study demonstrates the utility of controlled experiments to better understand how Pd viability varies among substrates, how persistence of Pd is potentially substrate specific, and how Pd can persist through time in different environmental reservoirs. Understanding the role of environmental substrates can help us to identify targets to apply management actions to prevent and control the indirect transmission of WNS via fomites.

## Supplementary Information

Supplementary Information

## Data Availability

Data are included as part of the supplementary information.

## References

[1] Fisher MC (2012). Emerging fungal threats to animal, plant and ecosystem health. Nature.

[2] Fisher MC, Gow NAR, Gurr SJ (2016). Tackling emerging fungal threats to animal health, food security and ecosystem resilience. Philos. Trans. R. Soc. B Biol. Sci..

[3] Ghosh PN, Fisher MC, Bates KA (2018). Diagnosing emerging fungal threats: A one health perspective. Front. Genet..

[4] Seyedmousavi S (2015). Aspergillus and aspergilloses in wild and domestic animals: A global health concern with parallels to human disease. Med. Mycol..

[5] Stephen C, Lester S, Black W, Fyfe M, Raverty S (2002). Multispecies outbreak of cryptococcosis on southern Vancouver Island, British Columbia. Can. Vet. J..

[6] Speare R, Thomas AD, O’Shea P, Shipton WA (1994). Mucor amphibiorum in the toad, Bufo marinus Australia. J. Wildl. Dis..

[7] Connolly JH (2009). A review of mucormycosis in the platypus (Ornithorhynchus anatinus). Aust. J. Zool..

[8] Gust N, Griffiths J (2009). Platypus mucormycosis and its conservation implications. Austral. Mycol..

[9] Thiel RP, Mech LD, Ruth GR, Archer JR, Kaufman L (1987). Blastomycosis in wild wolves. J. Wildl. Dis..

[10] Storms, T. N., Victoria L. Clyde, Linda Munson & Edward C. Ramsay. Blastomycosis in nondomestic felids. *J. Zool. Wildl. Med.* **34**, 231–238 (2003). 10.1638/1042-7260(2003)034[0231:BINF]2.0.CO;2 14582783

[11] Guillot, J., Guérin, C. & Chermette, R. Histoplasmosis in Animals. in *Emerging and Epizootic Fungal Infections in Animals* (eds. Seyedmousavi, S., de Hoog, G. S., Guillot, J. & Verweij, P. E.) 115–128 (Springer International Publishing, 2018). doi:10.1007/978-3-319-72093-7_5.

[12] Scheele BC (2019). Amphibian fungal panzootic causes catastrophic and ongoing loss of biodiversity. Science.

[13] Martel, A. *et al. Batrachochytrium salamandrivorans* sp. nov. causes lethal chytridiomycosis in amphibians. *Proc. Natl. Acad. Sci. USA* **110**, 15325 (2013). 10.1073/pnas.1307356110 PMC3780879 24003137

[14] Riley S (2007). Large-scale spatial-transmission models of infectious disease. Science.

[15] Johnson PTJ, de Roode JC, Fenton A (2015). Why infectious disease research needs community ecology. Science.

[16] Engering A, Hogerwerf L, Slingenbergh J (2013). Pathogen–host–environment interplay and disease emergence. Emerg. Microbes Infect..

[17] Shikano I, Cory JS (2015). Impact of environmental variation on host performance differs with pathogen identity: Implications for host-pathogen interactions in a changing climate. Sci. Rep..

[18] Kraay ANM (2018). Fomite-mediated transmission as a sufficient pathway: A comparative analysis across three viral pathogens. BMC Infect. Dis..

[19] Stephens B (2019). Microbial exchange via fomites and implications for human health. Curr. Pollut. Rep..

[20] Langwig, K. E. *et al.* Host and pathogen ecology drive the seasonal dynamics of a fungal disease, white-nose syndrome. *Proc. Biol. Sci.* **282**, (2015). 10.1098/rspb.2014.2335 PMC4286034 25473016

[21] Huebschman JJ (2019). Detection of Pseudogymnoascus destructans during Summer on Wisconsin Bats. J. Wildl. Dis..

[22] Hoyt JR (2020). Environmental reservoir dynamics predict global infection patterns and population impacts for the fungal disease white-nose syndrome. Proc. Natl. Acad. Sci. USA.

[23] Foley J, Clifford D, Castle K, Cryan P, Osfeld RS (2011). Investigating and managing the rapid emergence of white nose syndrome, a novel, fatal, infectious disease of hibernating bats. Conserv. Biol..

[24] Blanco CM, Garrie J (2020). Species specific effects of prescribed burns on bat occupancy in northwest Arkansas. For. Ecol. Manage..

[25] Gargas, A., Trest, M., Christensen, M., Volk, T. J. & Blehert, D. Geomyces destructans sp. nov. associated with bat white-nose syndrome. *Mycotaxon* **108**, 147–154 (2009).

[26] Blehert DS (2009). Bat white-nose syndrome: An emerging fungal pathogen?. Science.

[27] Cryan PM (2013). Electrolyte depletion in white-nose syndrome bats. J. Wildl. Dis..

[28] Warnecke L (2013). Pathophysiology of white-nose syndrome in bats: A mechanistic model linking wing damage to mortality. Biol. Lett..

[29] Verant ML (2014). White-nose syndrome initiates a cascade of physiologic disturbances in the hibernating bat host. BMC Physiol..

[30] Frick WF (2010). An emerging disease causes regional population collapse of a common North American bat species. Science.

[31] Turner, G. G., Reeder, D. M. & Coleman, J. T. H. A Five-year assessment of mortality and geographic spread of white-nose syndrome in North American Bats, with a Look at the Future. Update of white-nose syndrome in bats. *Bat Res. News* **52**, 13–27 (2011).

[32] Langwig KE (2012). Sociality, density-dependence and microclimates determine the persistence of populations suffering from a novel fungal disease, white-nose syndrome. Ecol. Lett..

[33] Langwig KE (2015). Invasion dynamics of white-nose syndrome fungus, midwestern United States. Emerg. Infect. Dis..

[34] USFW. U.S. Fish and Wildlife Service. 2019. White-nose syndrome: The devastating disease of hibernating bats in North America. Accessed 1 May 2020. https://www.whitenosesyndrome.org/mmedia-education/white-nose-syndrome-fact-sheet-june-2018. (2019).

[35] Lorch JM (2011). Experimental infection of bats with Geomyces destructans causes white-nose syndrome. Nature.

[36] Lorch JM (2013). Distribution and environmental persistence of the causative agent of white-nose syndrome, geomyces destructans, in bat hibernacula of the Eastern United States. Appl. Environ. Microbiol..

[37] Hoyt JR (2015). Long-term persistence of Pseudogymnoascus destructans, the Causative Agent of white-nose syndrome, in the absence of bats. EcoHealth.

[38] Campbell LJ, Walsh D, Blehert DS, Lorch JM (2020). Long-term survival of Pseudogymnoascus destructans at elevated temperatures. J. Wildl. Dis..

[39] Urbina J, Chestnut T, Schwalm D, Allen J, Levi T (2020). Experimental evaluation of genomic DNA degradation rates for the pathogen Pseudogymnoascus destructans (Pd) in bat guano. PeerJ.

[40] Lorch JM (2013). A culture-based survey of fungi in soil from bat hibernacula in the eastern United States and its implications for detection of Geomyces destructans, the causal agent of bat white-nose syndrome. Mycologia.

[41] Reynolds HT, Ingersoll T, Barton HA (2015). Modeling the environmental growth of Pseudogymnoascus destructans and its impact on the White-nose syndrome epidemic. J. Wildl. Dis..

[42] Warnecke L (2012). Inoculation of bats with European *Geomyces destructans* supports the novel pathogen hypothesis for the origin of white-nose syndrome. Proc. Natl. Acad. Sci. USA.

[43] WNS Multiagency decontamination team. https://www.whitenosesyndrome.org/mmedia-education/united-states-national-white-nose-syndrome-decontamination-protocol-april-2016-2. (2018).

[44] Verant M, Bohuski E, Lorch J, Blehert D (2016). Optimized methods for total nucleic acid extraction and quantification of the bat white-nose syndrome fungus, Pseudogymnoascus destructans, from swab and environmental samples. J. VET Diagn. Invest..

[45] Rocke TE (2019). Virally-vectored vaccine candidates against white-nose syndrome induce anti-fungal immune response in little brown bats (Myotis lucifugus). Sci. Rep..

[46] Zhelyazkova VL (2019). Screening and biosecurity for white-nose Fungus Pseudogymnoascus destructans (Ascomycota: Pseudeurotiaceae) in Hawai‘i. Pac. Sci..

[47] Muller LK (2013). Bat white-nose syndrome: A real-time TaqMan polymerase chain reaction test targeting the intergenic spacer region of Geomyces destructans. Mycologia.

[48] Vanderwolf KJ, Malloch D, McAlpine DF (2016). Detecting viable Pseudogymnoascus destructans (Ascomycota: Pseudeurotiaceae) from walls of bat hibernacula: Effect of culture media. J. Cave Karst Stud..

[49] Cheng TL (2017). Efficacy of a probiotic bacterium to treat bats affected by the disease white-nose syndrome. J. Appl. Ecol..

[50] Micalizzi EW, Mack JN, White GP, Avis TJ, Smith ML (2017). Microbial inhibitors of the fungus Pseudogymnoascus destructans, the causal agent of white-nose syndrome in bats. PLoS ONE.

[51] Singh A, Lasek-Nesselquist E, Chaturvedi V, Chaturvedi S (2018). Trichoderma polysporum selectively inhibits white-nose syndrome fungal pathogen Pseudogymnoascus destructans amidst soil microbes. Microbiome.

[52] De Mandal, S., Zothansanga, Panda, A. K., Bisht, S. S. & Senthil Kumar, N. First report of bacterial community from a Bat Guano using Illumina next-generation sequencing. *Genom. Data* **4**, 99–101. (2015). 10.1016/j.gdata.2015.04.001 PMC4535889 26484190

[53] Banskar S, Bhute SS, Suryavanshi MV, Punekar S, Shouche YS (2016). Microbiome analysis reveals the abundance of bacterial pathogens in Rousettus leschenaultii guano. Sci. Rep..

[54] Newman MM, Kloepper LN, Duncan M, McInroy JA, Kloepper JW (2018). Variation in bat guano bacterial community composition with depth. Front. Microbiol..

[55] Cruz MR, Graham CE, Gagliano BC, Lorenz MC, Garsin DA (2013). Enterococcus faecalis inhibits hyphal morphogenesis and virulence of Candida albicans. Infect. Immun..

[56] Graham CE, Cruz MR, Garsin DA, Lorenz MC (2017). Enterococcus faecalis bacteriocin EntV inhibits hyphal morphogenesis, biofilm formation, and virulence of Candida albicans. Proc. Natl. Acad. Sci. USA.

[57] Khan N (2018). Antifungal activity of bacillus species against fusarium and analysis of the potential mechanisms used in biocontrol. Front. Microbiol..

[58] Kerr JR (1999). Bacterial inhibition of fungal growth and pathogenicity. Microb. Ecol. Health Dis..

[59] Wheatley RE (2002). The consequences of volatile organic compound mediated bacterial and fungal interactions. Antonie Van Leeuwenhoek.

[60] Cornelison CT, Gabriel KT, Barlament C, Crow SA (2014). Inhibition of pseudogymnoascus destructans growth from conidia and mycelial extension by bacterially produced volatile organic compounds. Mycopathologia.

[61] Sussman A, Douthit H (1973). Dormancy in microbial spores. Annu. Rev. Plant Physiol..

[62] Feofilova, E. P., Ivashechkin, A. A., Alekhin, A. I. & Sergeeva, Ya. E. Fungal spores: Dormancy, germination, chemical composition, and role in biotechnology (review). *Appl. Biochem. Microbiol.* **48**, 1–11 (2012). 22567879

[63] Gasch AP (2007). Comparative genomics of the environmental stress response in ascomycete fungi. Yeast.

[64] Marroquin CM, Lavine JO, Windstam ST (2017). Effect of humidity on development of pseudogymnoascus destructans, the causal agent of bat white-nose syndrome. Northeastern Nat..

[65] Raudabaugh DB, Miller AN (2013). Nutritional capability of and substrate suitability for pseudogymnoascus destructans, the causal agent of bat white-nose syndrome. PLoS ONE.

[66] Gabriel KT, Kartforosh L, Crow SA, Cornelison CT (2018). Antimicrobial activity of essential oils against the fungal pathogens ascosphaera apis and pseudogymnoascus destructans. Mycopathologia.

[67] Boire N (2016). Potent inhibition of pseudogymnoascus destructans, the causative agent of white-nose syndrome in bats, by cold-pressed, terpeneless valencia orange oil. PLoS ONE.

[68] Turbill C, Welbergen JA (2020). Anticipating white-nose syndrome in the Southern Hemisphere: Widespread conditions favourable to Pseudogymnoascus destructans pose a serious risk to Australia’s bat fauna. Austral. Ecol..

