# Supplementary material for: Pseudogymnoascus destructans growth in wood, soil and guano substrates
Source: Sci Rep. 2021 Jan 12;11:763. doi: 10.1038/s41598-020-80707-1 (PMC7804951; doi:10.1038/s41598-020-80707-1)
Supplement: Supplementary file 1 — Supplementary Information [file 41598_2020_80707_MOESM1_ESM.pdf]

***Pseudogymnoascus destructans* growth in wood, soil and guano substrates**

Jenny Urbina [1*], Tara Chestnut [2], Jennifer M. Allen [1] and Taal Levi [1]

[1]*Department of Fisheries and Wildlife, Oregon State University, 2820 SW Campus Way, Nash Hall, Corvallis, OR, USA 97331*
[2]*Mount Rainier National Park, National Park Service, Ashford, WA, USA*

**Corresponding Author: jenny.gonzalez@oregonstate.edu*

SUPPLEMENTARY TABLE

Table 1 Amount of DNA (log10 (fg)) detected at 5 different sampling events in different substrates (Day 0, 16, 32, 48 and 64). The last two columns present information of different extraction kits used for wood.

Sampling event	Auto	Fresh	Soil	Wood PrepMan	Wood QIAGEN
**0**	4.93 ± 0.40	4.34 ± 0.87	4.62 ± 1.48	0.85 ± 1.45	NA
**1**	5.25 ± 0.23	4.83 ± 0.32	4.81 ± 0.16	0.78 ± 0.73	4.52 ± 0.15
**2**	4.92 ± 0.89	4.48 ± 0.80	4.08 ± 0.91	1.20 ± 0.75	4.46 ± 0.64
**3**	5.42 ± 0.19	4.39 ± 0.85	2.76 ± 0.47	0.34 ± 0.39	4.18 ± 0.09
**4**	6.04 ± 0.12	4.52 ± 0.99	2.87 ± 0.37	3.62 ± 1.32	5.12 ± 0.31
